# Characterisation of SalRAB a Salicylic Acid Inducible Positively Regulated Efflux System of *Rhizobium leguminosarum* bv *viciae* 3841

**DOI:** 10.1371/journal.pone.0103647

**Published:** 2014-08-18

**Authors:** Adrian J. Tett, Ramakrishnan Karunakaran, Philip S. Poole

**Affiliations:** 1 Department of Molecular Microbiology, John Innes Centre, Norwich, United Kingdom; 2 Department of Plant Sciences, University of Oxford, Oxford, United Kingdom; Technion-Israel Institute of Technology Haifa 32000 Israel, Israel

## Abstract

Salicylic acid is an important signalling molecule in plant-microbe defence and symbiosis. We analysed the transcriptional responses of the nitrogen fixing plant symbiont, *Rhizobium leguminosarum* bv *viciae* 3841 to salicylic acid. Two MFS-type multicomponent efflux systems were induced in response to salicylic acid, *rmrAB* and the hitherto undescribed system *salRAB*. Based on sequence similarity *salA* and *salB* encode a membrane fusion and inner membrane protein respectively. *salAB* are positively regulated by the LysR regulator SalR. Disruption of *salA* significantly increased the sensitivity of the mutant to salicylic acid, while disruption of *rmrA* did not. A *salA/rmrA* double mutation did not have increased sensitivity relative to the *salA* mutant. Pea plants nodulated by *salA* or *rmrA* strains did not have altered nodule number or nitrogen fixation rates, consistent with weak expression of *salA* in the rhizosphere and in nodule bacteria. However, BLAST analysis revealed seventeen putative efflux systems in Rlv3841 and several of these were highly differentially expressed during rhizosphere colonisation, host infection and bacteroid differentiation. This suggests they have an integral role in symbiosis with host plants.

## Introduction

Plants produce and secrete a diverse number of compounds into the rhizosphere. These include a myriad of phytoalexins and signalling molecules which not only mediate plant defences but orchestrate plant microbial interactions including symbiosis with nitrogen fixing symbiotic rhizobia [1], [2]. One key molecule in the response of plants to microbes is salicylic acid, which is a phenolic hormone with varied roles in plant metabolism and physiology including plant defence [3]. When a plant recognises a biotrophic pathogen, salicylic acid regulates the specific and localised Hypersensitive Response (HR) leading to cell death at the site of infection [4]. Salicylic acid is also involved in the longer lasting systemic protection of the plant against a range of pathogens, termed Systemic Acquired Resistance (SAR) [5]. Plants and microbes have evolved complex signal interactions in order to distinguish friend from foe. In the case of symbioses with rhizobia plants do not usually elicit a defence response [6], although salicylic acid may be important in controlling host range and regulating nodule formation. A number of studies have investigated salicylic acid levels and nodule formation. Exogenous application of salicylic acid has been shown to decrease or inhibit nodule formation when *Bradyrhizobium japonicum* or *Rhizobium leguminosarum* are grown on Soybean and Vetch respectively [7], [8]. Similarly, decreasing endogenous levels of salicylic acid in *Lotus japonicus* led to increased nodule numbers when inoculated with *Mesorhizobium loti* [6].

One mechanism to circumvent the toxic effects of compounds that microbes encounter in the environment is their extrusion via efflux systems. These systems are ubiquitous in bacteria and comprise five superfamilies [9]. The first belong to the ATP Binding Cassette (ABC) family, which are primary transporters using ATP hydrolysis to drive efflux. The other four, The Major Facilitator Superfamily (MFS), the Resistance Nodulation Division (RND), the Multi Antimicrobial Extrusion (MATE) and Drug Metabolite Transporters (DMT)/SMR are secondary H^+^ or Na^+^ antiporters. In gram negative bacteria some ABC, MFS and all RND are multicomponent tripartite systems spanning both inner and outer membranes. They comprise an inner membrane transporter, a membrane fusion protein and a TolC like outer membrane factor [10]. Usually, the inner membrane transport and membrane fusion protein are located together however, cells generally have several TolC like proteins that act with a number of different efflux systems [10].

Successful phytopathogenic bacteria must export or detoxify plant phytoalexins, with disruption of two RND type efflux systems in *Erwinia amylovora* (ArcAB) and *Pseudomonas syringae* (MexAB) reducing virulence on apple trees and bean leaves respectively [11], [12]. The role of these systems in promoting pathogenesis is clear but efflux systems are also common in plant symbiotic bacteria such as the nitrogen fixing rhizobia. Disruption of the multicomponent MFS efflux pump (*rmrAB*) in *Rhizobium etli* CFN42 increased phytoalexin sensitivity and led to impaired nodule formation. [13]. Likewise, nitrogen fixation was impaired and antimicrobial sensitivity increased when the RND family *bdeAB* efflux system was disrupted in the soybean symbiont *Bradyrhizobium japonicum* 110*spc*4 [14]. Nodulation, competiveness and toxin sensitivity were also affected when the RND *smeAB* pump was disrupted in *Sinorhizobium meliloti* 1021 [15].

We investigated the transcriptional responses of *Rhizobium leguminosarum* bv *viciae* 3841 to salicylic acid, coupled with a genome screen to identify putative multicomponent efflux systems. We investigated the contribution of these systems to salicylic acid resistance and their induction during plant colonisation and nodulation.

## Results

### *R. leguminosarum* 3841 transcriptional responses to salicylic acid

**Figure 1:**
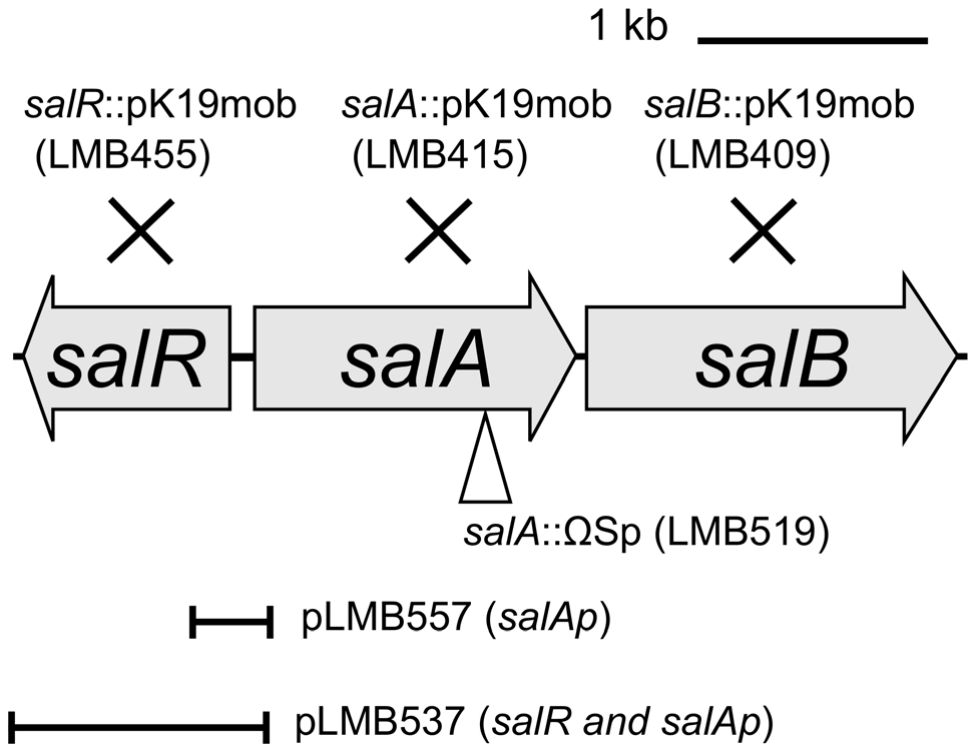
Schematic diagram of the Rlv3841 *salRAB* operon. Genomic organisation of the *salRAB* genes; *salR* encodes a putative LysR like regulator and *salA* and *salB* a putative membrane fusion protein and an inner membrane transporter respectively. Site of *salA* deletion (LMB519) and independent *salRAB* pK19mob insertion mutants (LMB455, LMB415 and LMB409) used in this study are shown. Genomic fragments used to construct transcriptional reporter plasmids pLMB557 (*salA* promoter only) and pLMB537 (*salA* promoter as well as *salR*) are also depicted.

In response to the addition of salicylic acid (0.72 mM) a total of 21 genes were up-regulated more than two fold (t-test p≤0.05) compared to free living cells (Table 1). These responses can be broadly classified into those likely to be involved in salicylic acid export or its catabolism. Two genes RL1329 and RL1330 encoding putative efflux pump components were upregulated 18.1 and 2.2 fold respectively, with RL1329 being the most highly elevated gene in Rlv3841 in response to salicylic acid. Upstream of these genes is a putative LysR like transcriptional regulator, RL1328. It is proposed these genes be designated *salR* (RL1328), *salA* (RL1329) and *salB* (RL1330) (Figure 1). Based on homology with known proteins it is proposed *salRAB* is a MFS family multicomponent efflux system, where *salA* is a putative membrane fusion protein and *salB* is an inner membrane transporter. In addition *rmrA* (pRL90059), which has 85% identity to the membrane fusion protein of the characterised MFS family efflux pump in *R. etli* CFN42 [13], was also upregulated 4.1 fold.

**Table 1:** Rlv3841 genes above two fold upregulated in response to 0.72 mM salicylic acid.

Gene id [31]	Fold induction	P-value	Gene name	Product description [31]
pRL110531	4.2	0.0012		hypothetical protein
pRL110532	6.9	0.0009		conserved hypothetical protein
pRL110534	5.4	0.0598		putative two-component sensor kinase regulator
pRL110535	2.2	0.1101		putative two-component response regulator
pRL110536	2.9	0.0055		putative haloalkane dehalogenase
pRL120214	2.0	0.0002		putative dioxygenase
pRL120215	2.1	0.1313	*tftE1*	putative maleylacetate reductase
pRL120216	6.0	0.0231		putative amidohydrolase
pRL120218	2.3	0.1362		putative flavin reductase
pRL120616	2.1	0.0081		putative reductase
pRL90059	4.1	0.0233	*rmrA*	putative type I HlyD transporter
RL0272	4.7	0.0017		putative aldo-keto reductase/oxidoreductase
RL0577	3.8	0.0001		putative transmembrane protein
RL1329	18.1	0.0051	*salA*	putative HlyD family efflux pump protein
RL1330	2.2	0.0189	*salB*	putative transmembrane efflux pump protein
RL1507	2.2	0.0288		conserved hypothetical protein
RL1860	8.6	0.0000	*phhA*	putative phenylalanine-4-hydroxylase
RL1910	2.2	0.0549		conserved hypothetical protein
RL1911	7.7	0.0312		putative arylsulfatase
RL1917	2.7	0.0718		conserved hypothetical protein
RL1918	4.3	0.0859		putative exported arylsulfatase protein
RL1924	3.4	0.1454		conserved hypothetical exported protein
RL1925	4.1	0.1013		conserved hypothetical protein
RL1966	4.6	0.0977	*aldA*	alanine dehydrogenase
RL2029	2.2	0.0056		hypothetical protein
RL2670	2.9	0.0482		putative oxidoreductase
RL2726	2.5	0.0003	*ribA1*	putative riboflavin biosynthesis protein
RL3172	2.9	0.0061		hypothetical protein
RL3286	3.3	0.0003		putative ABC transporter component, pseudogene
RL3297	2.0	0.0067	*lpxC*	putative UDP-3-O-[3-hydroxymyristoyl] N-acetylglucosamine deacetylase
RL4612	2.3	0.0006		putative transmembrane MFS family transport

### Mutation of the *sal* genes

**Figure 2:**
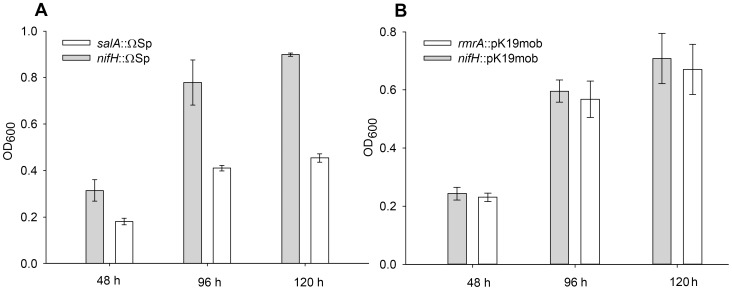
Salicylic acid sensitivity assays. **A,** Single *salA* mutant, *salA*::ΩSp (LMB519) and wild type control *nifH*::ΩSp (RU3940) grown in AMS and 2.0 mM salicylic acid. **B,** Single *rmrA* mutant, *rmrA*::pK19mob (RU4314) and wild type control, *nifH*::pK19mob (RU4062) grown in AMS and 2.0 mM salicylic acid. Data are shown as the mean ± standard error of the mean (SEM) for triplicate cultures.

To determine if disruption of the *salRAB* operon in *R. leguminosarum* 3841 alters sensitivity to salicylic acid a *salA* deletion mutant was isolated (LMB519) and growth assayed at varying salicylic acid concentrations. In addition, as loss of *rmrA* has been shown to increase salicylic acid sensitivity in *R. etli* CFN42 [13], a Rlv3841 *rmrA* (pRL90059) deletion mutant previously isolated [16] was also tested. Salicylic acid (2 mM) significantly impaired growth of the *salA* mutant compared to controls (Figure 2A). In contrast disruption of *rmrA* led to no detectable difference in growth compared to wild type controls (Figure 2B). Furthermore a double *salA/rmrA* (LMB523) mutant was not more sensitive to salicylic acid than the single *salA* mutant. In this instance the appropriate wild type control for comparison to the *salA*/*rmrA* (LMB523) mutant was RU4223 [16] which contains a ΩSp and pK19mob mutation in genes unrelated *salRAB*. This was so appropriate antibiotic selection could be used in the media and any differences in growth due to the presence of the spectinomycin cassette or pK19mob insertion could be discounted.

**Figure 3:**
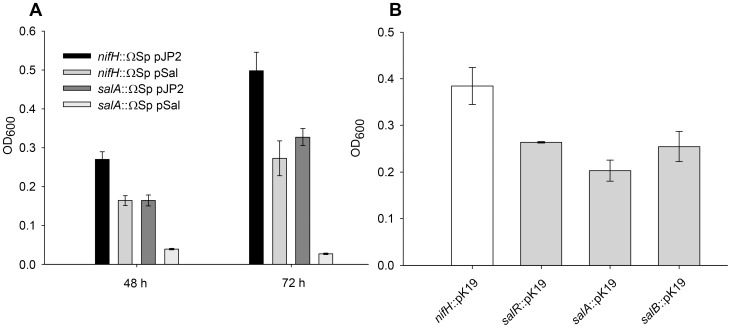
Complementation assay and *salRAB* independent insertion mutations. **A,** Growth of *nifH*::ΩSp (RU3940) and *salA*::ΩSp (LMB519), in AMS and 2.0 mM salicylic acid, when carrying either pJP2 control or pSal containing the full *salRAB* operon. **B,** Independent pK19 insertion mutations of the three genes of the *salRAB* operon compared to wild type control (*nifH*::pK19mob) when grown for 48 hours in AMS and 1.45 mM salicylic acid. Data are shown as the mean ± standard error of the mean (SEM) for triplicate cultures.

In order to complement the *salA* gene disruption the whole Sal operon (*salRAB*) was cloned into the stable low copy number plasmid pJP2 [17] forming pSal. When not induced with salicylic acid carriage of pSal had no effect on growth of either *salA*::ΩSp (LMB519) or WT control *nifH*::ΩSp (RU3940) compared to pJP2 parent plasmid containing strains (LMB641 and LMB640 respectively). A *nifH* mutant was used as a control instead of Rlv3841 because it enabled spectinomycin to be included in all media, ensuring growth differences were not due to the presence of a ΩSp cassette or spectinomycin. When induced with salicylic acid, instead of complementing the *salA*::ΩSp mutation (LMB519) pSal reduced growth relative to a strain (LMB641) containing the parent plasmid pJP2 alone. Similarly, pSal in the wild type control RU3940 (*nifH*::ΩSp) also reduced growth relative to pJP2 containing strain (LMB640) when induced with salicylic acid (Figure 3A). Since salicylic acid is required for induction of the *sal* operon (see below) this suggests over-expression of the *salAB* operon from a low copy number plasmid is inhibitory and reduces growth. The problem of over expression of a membrane transporter leading to reduced growth is not uncommon. Since complementation proved problematic in order to confirm that the disruption of the *salA* was responsible for increased sensitivity to salicylic acid, independent pK19mob insertion mutants were isolated for each gene of the Sal operon. In the absence of salicylic acid there was no growth difference between wild type and mutant strains (Doubling times (h ± standard error of the mean for three triplicates); RU4062 (*nifH*::pK19mob) 8.3±0.017, LMB455 (*salR*::pK19mob) 8.2±0.06, LMB415 (*salA*::pK19mob) 8±0.2, LMB409 (*salB*::pK19mob) 8±0.03). With the addition of 1.45 mM salicylic acid all three *sal* mutants had reduced growth compared to wild type strain (*nifH*::pK19mob) (Figure 3B) as was observed with LMB519 (*salA*::ΩSp) (Figure 2A).

### Plant symbiosis and antimicrobial sensitivity

The disruption of multicomponent efflux systems in other rhizobia has been shown to affect sensitivity to toxins, nodule formation and/or nitrogen fixation activity [13]–[15]. To ascertain if disruption of either the *salRAB* or *rmrAB* affected plant nodulation and nitrogen fixation the *salA* (LMB519), *rmrA* (RU4314) and double *salA*/*salR* (LMB523) mutants were inoculated on pea seeds. After 21 days growth the number of nodules were recorded and nitrogen fixing activity assessed by acetylene reduction assays. Compared to wild type none of the mutants differed in either the number of nodules formed (Rlv3841 (WT) 105±6.0; LMB519 (*salA*::ΩSp) 110±7.3; RU4314 (*rmrA*::pK19mob) 109±6.4; LMB523 (*salA*::ΩSp/*rmrA*::pK19mob) 114±4.7) or in nitrogen fixation (ARA (µmoles ethylene/plant/h) Rlv3841 (WT) 13.7±0.8; LMB519 (*salA*::ΩSp) 14.5±0.9; RU4314 (*rmrA*::pK19mob) 14.9±1.4; LMB523 (*salA*::ΩSp/*rmrA*::pK19mob) 15.6±1.1). In addition, the mutants did not have increased sensitivity to the antibiotics tetracycline and Nalidixic acid. It has been reported that disruption of efflux systems in other rhizobia, including *rmrA* mutant strains of CFN42, led to increased sensitivity to flavonoids and alkaloids [13], [15]. However, neither Rlv3841 (WT), *salA* (LMB519), *rmrA* (RU4314) nor *salA*/*rmrA* (LMB523) strains of Rlv3841 showed impaired growth on agar plates with filter discs of genistein, naringenin or Berberine.

### *salRAB* regulation

**Figure 4:**
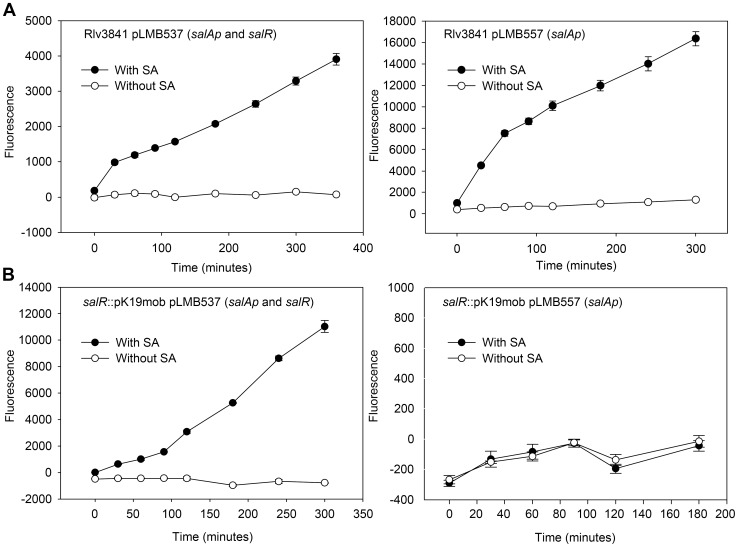
Transcription reporter assays. **A,** Rlv3841 wild type carrying transcriptional fusion plasmid pLMB537 (containing *salAp* and *salR*) or pLMB557 (*salAp* only), either un-induced of induced with 0.72 mM salicylic acid (SA). **B,** LMB455 (Rlv3841 *salR*::pK19mob) carrying pLMB537 (containing *salAp* and *SalR*) or pLMB557 (*salAp* only) either un-induced of induced with 0.72 mM salicylic acid (SA). Fluorescence of GFP mut3.1 reporter protein was detected by Tecan GENios fluorometer (excitation 485 nm, emission 510 nm). Data are shown as the mean ± standard error of the mean (SEM) for triplicate assays.

To examine the regulation of the *salRAB* operon two transcriptional reporter plasmids were constructed. Plasmid pLMB557 contains the putative intergenic promoter region between *salR* and *salA* (*salAp*) (Figure 1) upstream of a promoterless *gfpmut3.1* reporter. The second plasmid (pLMB537) contains the promoter region as well as the complete *salR* gene (*salAp* and *salR*) (Figure 1). The plasmids were introduced into the mutant strain LMB455 (*salR*::pK19mob) and Rlv3841. In the absence of salicylic acid there was no detectable induction of *salA* (Figure 4). When Rlv3841 (WT) carrying pLMB537 (*salA*p and *salR*) or pLMB557 (*salAp* only) were incubated with 0.72 mM salicylic acid *salA* was induced (Figure 4A). However, *salA* was not induced in the *salR* insertion mutant (LMB455) containing pLMB557 (*salAp* only) (Figure 4B). This indicates that the *salRAB* operon is positively regulated by SalR. This was confirmed by restoration of *salA* induction in the *salR* insertion mutant (LMB455) when carrying pLMB537, which contains *salAp* and a full length copy of *salR* (Figure 4B).

### Induction specificity of the *salRAB* operon

It is proposed that salicylic acid is synthesised in plants via two enzymatic pathways from the primary metabolite chorismate, either via phenylalanine, cinnamate and benzoate intermediaries, or by a second pathway from isochorismate [18]. To test the specificity of the Sal operon several of these proposed intermediates and derivatives were used in induction assays with Rlv3841 containing the reporter plasmid pLMB557 (LMB475). These included cinnamate, benzoate and phenylalanine as well as the volatile ester of salicylic acid, methyl salicylate (wintergreen oil), a plant defence signalling molecule [19]. In addition catechol, p-hydroxybenzoate, protocatechuic acid and acetylsalicylic acid (aspirin) that have a similar structure to salicylic acid were also tested. Apart from salicylic acid itself all of these compounds failed to induce pLMB557, except for acetylsalicylic acid which showed some induction after 24 hours. This slow and weak induction may be the result of spontaneous hydrolysis of acetylsalicylic acid releasing salicylic acid.

### Other multicomponent efflux systems of Rlv3841

To put the MFS type *salRAB* and *rmrAB* in context of the total multicomponent efflux systems encoded by Rlv3841, the genome was screened for proteins with sequence similarity to characterised systems. In total 17 systems were identified, including *salRAB* and *rmrAB* (Table 2). Eleven of the systems belong to the RND family, four to the MFS family and two to the ABC families. Fifteen of the systems were encoded on the chromosome and one each on pRL9 and pRL10. In addition several TolC like homologs were also identified (RL3876, pR100291 and pR100178).

**Table 2:** Putative multicomponent efflux systems of *R. leguminosarum* bv *viciae* 3841.

Designated family	IMT	MFP	Replicon	Sequence position
MFS	pRL90060 (*rmrB*)	pRL90059 (*rmrA*)	pRL9	61508..64307
RND	RL3875 (*rmeB*)	RL3874 (*rmeA*)	Chromosome	4098266..4102636
RND*	pRL100286	pRL100287	pRL10	295910..300156
MFS	RL1330 (*salB*)	RL1329 (*salA*)	Chromosome	1392779..1395765
RND*	RL1453	RL1454	Chromosome	1516274..1520694
ABC*	RL2365	RL2364	Chromosome	2485492..2487513
RND	RL2666	RL2667	Chromosome	2814549..2818924
RND	RL3725	RL3724	Chromosome	3921000..3925529
RND*	RL3774	RL3775	Chromosome	3978570..3983080
RND	RL4223	RL4224	Chromosome	4475300..4479612
RND*	RL4275	RL4274	Chromosome	4531632..4536139
RND	RL3787	RL3786	Chromosome	3992927..3997287
MFS	RL3783	RL3784	Chromosome	3989164..3991792
RND	pRL120698	pRL120696/pRL120697	Chromosome	752595..757818
ABC	RL3029	RL3030	Chromosome	3193055..3194982
RND*	RL3269	RL3270	Chromosome	3421636..3425996
MFS	RL4179	RL4180	Chromosome	4430233..4433070

Abbreviations: IMT, inner membrane protein; MFP, membrane fusion protein. * No adjacent transcriptional regulator.

**Figure 5:**
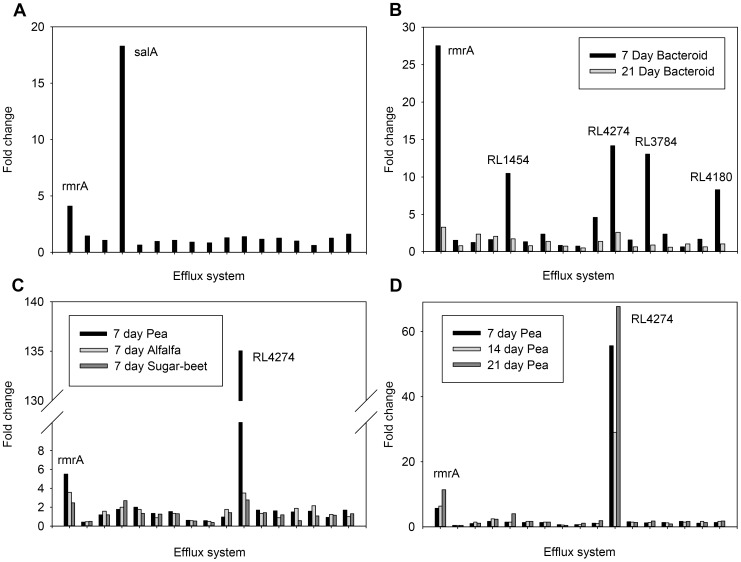
Transcriptional responses of Rlv3841 putative tripartite efflux systems to different environments determined by microarray analysis. Efflux systems are in order as given in Table 2. Fold changes are given for the membrane fusion protein (MFP) of each efflux system. **A,** Addition of 0.72 mM salicylic acid. **B,** Bacteroids isolated from 7 and 21 day nodules [16]. **C,** Rlv3841 isolated 7 day post inoculation from 7 day old pea (*Pisum sativum*), alfalfa (*Medicago sativa*) and sugar beet (*Beta vulgaris*) after 7 day post inoculation [20]. **D,** Rlv3841 isolated 1 day post inoculation from 7, 14 and 21 day old peas [20].

We have collected a large dataset (73 growth conditions) of the transcriptional response of Rlv3841 to different environments and inducers, most of which has been published previously, including colonisation of different plant rhizospheres [20] and in bacteroid development [16]. This allowed analysis of the expression of the efflux transporters when interacting with host plants, as well as in response to salicylic acid (Figure 5). In early seven day bacteroid development many of the systems were highly upregulated compared to free living cells and in comparison to 21 day bacteroids (Figure 5B). In total five systems show an above five-fold elevation at seven days with *rmrA* the highest at 27.5 fold up. In contrast *salA* has a modest 1.5 fold change. On colonisation of the rhizosphere of seven-day old pea, alfalfa and sugar beet (Figure 5C) both *rmrA* and *salA* showed transcriptional increases of 5.5,3.5,2.5- and 1.8,2,2.7-fold, respectively. By far the largest increase at 135-fold was that of RL4274, a putative RND type efflux pump. How the efflux systems are differentially expressed when Rlv3841 colonises Pea plants of different ages is given in Figure 5D. Thus *salRAB* is only weakly induced in the plant rhizosphere and during nodule formation explaining the lack of a phenotype in a *salA* mutant.

## Discussion

Multidrug efflux systems are important to plant pathogens and symbionts alike. Regardless of lifestyle soil organisms must overcome both abiotic and biotic stresses, including antimicrobials derived from plants and microbial competitors. In this study we investigated the global transcriptional response of Rlv3841 to salicylic acid, and identified two efflux systems significantly unregulated. The first of these was the hitherto uncharacterised *salRAB* and the second *rmrAB*, which has 85% (*rmrA*) and 89% (*rrmB*) amino acid identity to the characterised efflux system of *R. etli* CFN42 [13].

Disruption of *salRAB* in Rlv3841 led to significantly increased sensitivity to salicylic acid. However, it did not increase sensitivity to other antimicrobials tested or affect nodulation and nitrogen fixation. In *R. etli* CFN42, a close relative of Rlv3841 which also shows a high degree of sequence similarity to the *salRAB* system of Rlv3841 (RHE_CH01191, 90%; RHE_CH01192, 82% and RHE_CH01193 93% amino acid similarity to *salRAB* respectively), it has been demonstrated that disruption of *rmrA* leads to a 40% decrease in nodule number on bean (*Phaseolus vulgaris*) and five-fold increase in sensitivity to salicylic acid [13]. In contrast, disruption of *rmrA* in Rlv3841 did not increase sensitivity to salicylic acid or other antimicrobials. Furthermore, a double *rmrA salA* mutant did not have greater sensitivity to salicylic acid than the *salA* mutant. One explanation for the difference between the two organisms could be functional redundancy where efflux systems and/or other translocation mechanisms might overlap in substrate specificity. This is particularly true of soil organisms, which contain a disproportionally high number of efflux systems [21]. Thus it is possible the resilience of Rlv3841 to salicylic acid could suggest an as of yet unknown determinant that partially compensates the loss of both *salRAB* and *rmrAB*.

In addition to functional redundancy there are inherent difficulties in functional characterisation of efflux systems. Due to the wide diversity of compounds translocated by these systems, identifying the appropriate compounds is challenging. Efflux systems can also be host specific, for example disruption of the RND type efflux system *bdeAB* of *Bradyrhizobium japonicum* 110spc4 led to a decrease in the number of bacteroids in nodules and in nitrogen fixation when grown on soybean, but not on other plant hosts, mungbean and cowpea [14]. In addition, not all efflux may be responsible for removal of antimicrobials from cells, for example in *Pseudomonas aeruginosa* the MexAB-OprM system is involved in transport of compounds for quorum sensing [22], therefore their disruption is unlikely to affect antimicrobial resistance. In a recent study 14 multicomponent efflux systems were identified in *S. meliloti* 1021 via screening the genome for genes similar to known efflux pump components [15]. Each of these systems was deleted individually. Of the 14 mutants only one, *smeAB*, led to an increase in antimicrobial sensitivity and loss of competitive colonisation fitness compared to wild type. Double mutations of *smeAB* and systems *smeCD* and *smeEF* led to further antimicrobial sensitivity, however for 11 systems no difference in antimicrobial susceptibility was detected.

In this study we identified 17 putative efflux systems in Rlv3841 and interrogated transcriptional data previously produced from our lab to gain insight into the induction of these systems during rhizosphere colonisation, host infection and bacteroid differentiation during nodulation. In seven day bacteroids over one third of all the putative encoded efflux systems were induced above three-fold, after 21 days only one system was induced above three-fold. The involvement of so many systems is testament to the complex and temporal physiological conditions encountered during nodule development. It is also possible that these systems overlap in specificity and thus offer an explanation why disruption of *rmrA*, the most uprgeulated system in seven-day bacteroids, had no effect on nodulation and nitrogen fixation. Furthermore, many of these systems may be expressed constitutively resulting in significant background resistance to antimicrobials.

Similarly, during plant colonisation of pea, alfalfa and sugar-beet a large number of different efflux systems were up regulated including *salRAB* and *rmrAB*. In addition most respond similarly to the different rhizospheres i.e. are generalists. Although, one system (RND type RL4274/4275), while upregulated in all three rhizospheres, was most highly induced by pea (Figure 5C), suggesting specific induction for this system. Indeed in a previous study mutation of RL4274 decreased the competiveness compared to Rlv3841 in the pea rhizosphere [20].

In this study we have demonstrated that *salAB* is positively regulated by a LysR family transcriptional type regulator SalR. Induction of this operon is highly specific to salicylic acid. Salicylic acid is known to be instrumental in nodule development such that in *Rhizobium leguminosarum* bv *viciae* strains RBL 5523 and 248 exogenous application of 10^−4^ M salicylic acid completely inhibited nodule formation on vetch [8]. Moreover, Stacey et al., 2006 [6] demonstrated that reducing the endogenous levels of salicylic acid by transgenic expression of salicylate hydroxylase (NahG) in *Lotus japonicus* correlated with an increase in nodule number when inoculated with *Mesorhizobium loti*. As *salRAB* confers increased resistance at high levels of salicylic acid (above 1.45 mM) it can be hypothesised *salRAB* confers a fitness advantage to Rlv3841 by eliminating the inhibitory effects of salicylic acid through expulsion from the cell. However, the Sal system was only weakly expressed in the rhizosphere and in nodule bacteria explaining the lack of effect of mutation in *salA* on nodulation and N_2_-fixation.

## Materials and Methods

### Strains, plasmids and culture conditions

The strains and plasmids used in this study are detailed in Table 3. All *Escherichia coli* strains were grown at 37°C in Lennox (L) broth or L agar. *R. leguminosarum* strains were grown at 28°C in Tryptone-yeast extract [23] or Acid Minimal Salts (AMS) [24] supplemented with 30 mM Pyruvate and 10 mM ammonium chloride and agitated at 220 rpm. Antibiotics were used in the following concentrations (µg ml^−1^) unless otherwise stated. Gentamicin, 20 (10 in *E. coli*); Kanamycin, 20; Neomycin, 20; Spectinomycin, 50; Streptomycin 500; Tetracycline (2 in AMS, 5 in TY).

**Table 3:** Bacterial strains and plasmids used in this study.

Strain or plasmid	Description	Source or reference
**Plasmid**		
pHP45Ω	pBR322 derivative carrying ΩpHP45 replicon; Ap^r^ Sp^r^	[27]
pJET1.2 Blunt	PCR product cloning vector; Ap^r^	Thermo Scientific
pJP2	Stable broad host range cloning vector; Tc^r^	[17]
pJQ200SK	Suicide vector *sacB* gene; Gm^r^	[28]
pK19mob	mob+; Km^r^	[32]
pLMB474	pK19 containing internal fragment of *salB*/RL1330; Km^r^	This work
pLMB492	pK19 containing internal fragment of *salA*/RL1329; Km^r^	This work
pLMB537	pRU1097 parent transcriptional fusion WITH regulator *salR*/RL1328 and *salA* promoter region (*salAp and salR*); Gm^r^	This work
pLMB546	pK19 containing internal fragment of *salR*/RL1328; Km^r^	This work
pLMB557	pRU1097 parent transcriptional fusion WITHOUT regulator *salR*/RL1328 only the salA promoter region (*salAp*); Gm^r^	This work
pLMB607	pr1310-pr1311 PCR product in pJET1.2 blunt; Ap^r^	This work
pLMB608	ΩSp cassette cloned into EcoRI site of pLMB607; Ap^r^ Sp^r^	This work
pLMB611	XbaI/XhoI fragment from pLMB608 cloned into pJQ200SK; Ap^r^ Sp^r^	This work
pRK2013	ColEI replicon with RK2 *tra* genes, a helper plasmid used for mobilising plasmids; Km^r^	[33]
pRU1097	Promoterless *gfpmut3.1* cloning vector; Gm^r^	[34]
pSal	pJP2 parent, complete *salRAB* operon (RL1328-1330); Tc^r^	This work
***R. leguminosarum***		
Rlv3841	Str^r^ derivative of *R. leguminosarum* 300	[35]
LMB409	Rlv3841 *salB*/RL1330::pK19mob (pLMB492)	This work
LMB415	Rlv3841 *salA*/RL1329::pK19mob (pLMB546)	This work
LMB455	Rlv3841 *salR*/RL1328::pK19mob (pLMB474)	This work
LMB475	Rlv3841 with pLMB557	This work
LMB519	Rlv3841 *salA*::ΩSp cassette	This work
LMB523	LMB519 (*salA*/RL1329::ΩSp cassette); *rmrA*::pK19mob	This work
LMB635	RU3940 (*nifH*::ΩSp cassette) with pSal	This work
LMB636	LMB519 (*salA*/RL1329::ΩSp cassette) with pSal	This work
LMB637	LMB523 (*salA*/RL1329::ΩSp cassette; *rmrA*::pK19mob) with pSal	This work
LMB640	RU3940 (*nifH*::ΩSp cassette) with pJP2	This work
LMB641	LMB519 (*salA*/RL1329::ΩSp cassette) with pJP2	This work
LMB649	Rlv3841 with pLMB537	This work
LMB650	LMB455 (*salR*/RL1328::pK19mob) with pLMB537	This work
LMB651	LMB455 (*salR*/RL1328::pK19mob)with pLMB557	This work
RU3940	Rlv3841 *nifH*::ΩSp cassette	[16]
RU4062	Rlv3841 *nifH*::pK19mob	[16]
RU4223	Double glucose transporter mutant	[16]
RU4314	Rlv3841 *rmrA*::pK19mob	[16]

Abbreviations: Ap^r^, ampicillin; Gm^r^, gentamicin; Km^r^, Kanamycin; Sp^r^, spectinomycin; Str^r^, streptomycin; Tc^r^, tetracycline.

### Molecular Microbiology

All general DNA cloning was as described [25]. PCR amplification, unless otherwise stated, was performed in 100 µl volumes using 2.5 units GoTaq (Promega), 1 µM primer and 0.2 mM dNTPs. Cycling conditions were: One cycle of 95°C for 2 minutes, 30 cycles of 95°C for 45 s, 57°C for 45 s, 72°C for 3 minutes with a final extension of 10 minutes at 72°C. All constructs were confirmed using Sanger sequencing. Plasmids were conjugated from *E. coli* into *R. leguminosarum* strains using the helper plasmid pRK2013 as previously described [26]. PCR primers used in this study are given in Table 4.

**Table 4:** Primers used in this study.

Primer name	Sequence	Function
RL1328_BD_F	TGATTACGCCAAGCTCCGAACTCGATGAGGCGGAC	To amplify internal fragment of *salR* (RL1328)
RL1328_BD_R	GCAGGCATGCAAGCTTTCGGCGATGTCGTCGAACA	To amplify internal fragment of *salR* (RL1328)
RL1328_MAP	TCATTGTCGTTGACGAGCAC	*salR* (RL1328) pK19 mapping primer
RL1329_BD_F	TGATTACGCCAAGCTGTGCGAGGGAGGCTGAGCAGAT	To amplify internal fragment of *salA* (RL1329)
RL1329_BD_R	GCAGGCATGCAAGCTTTGGCCTGCTCGATCACCGATT	To amplify internal fragment of *salA* (RL1329)
RL1329_MAP	CGACGGGCAGCAGGAAACGC	*salA* (RL1329) pK19 mapping primer
RL1330_BD_F	TGATTACGCCAAGCTCGATGACGGCGGCTGGATTTC	To amplify internal fragment of *salB* (RL1330)
RL1330_BD_R	GCAGGCATGCAAGCTTCCTTGTTGCCCTCTTCCAGCA	To amplify internal fragment of *salB* (RL1330)
RL1330_MAP	CTCGCTGCTGCCGCCGGACA	*salB* (RL1330) pK19 mapping primer
pK19A	ATCAGATCTTGATCCCCTGC	pK19 mapping primer
pK19B	GCACGAGGGAGCTTCCAGGG	pK19 mapping primer
Pr1310	TTTTCTAGAATAGACCTTCAGCGTGCCCC	To amplify *salRAB* operon
Pr1311	TTTCTCGAGTGTGCACGTTCATGAAGTTC	To amplify *salRAB* operon
Pr1312	GTCTTCGGCATCGGATGGCT	Sequencing primer to confirm spectinomycin cassette insertion in *salA* (RL1329)
Pr1394	TTGGTACCTACCCCTCACGTCATCCACT	To amplify *salRAB* operon
Pr1395	TTGGATCCTTCTCCTCCTTGAGGGTCGC	To amplify *salRAB* operon
PotFarForward	GACCTTTTGAATGACCTTTA	Sequencing primer to confirm spectinomycin cassette insertion in *salA* (RL1329)
E6_RL1328_for	TTTAAGCTTGAAGGGTGACCTCCAATGTA	To amplify *salR* (RL1328) gene and promoter region for creating pLMB537
E6_RL1328_rev	TTTGGATCCCAGCCTCCCTCGCACTTTCG	To amplify *salR* (RL1328) gene and promoter region for creating pLMB537
E6_P_For	TTTAAGCTTGCCGACCATGGTGACGG	To amplify promoter region upstream of *salA* (RL1329) for creating pLMB557
E6_P_rev	TTTGGATCGGCGCTTCGGAGACAGGC	To amplify promoter region upstream of *salA* (RL1329) for creating pLMB557

### RNA isolation and microarray analysis

*R. leguminosarum* 3841 was grown in triplicate in 100 ml AMS to mid-log growth phase before the cells where harvested and washed twice in AMS and resuspended in AMS with or without 0.72 mM salicylic acid (i.e. 100 µg ml^−1^). Cultures were induced for three hours before RNA was extracted, amplified and hybridised as previously described [16]. Microarray data are available in the ArrayExpress database (www.ebi.ac.uk/arrayexpress) under accession number E-MTAB-2187.

### Induction reporter assays

Induction assays were performed in 100 ml volumes. Cells were grown to mid log growth phase before candidate inducers were added to the media to a final concentration of 0.72 mM. Assays were performed in triplicate and GFP fluorescence was detected with a Tecan GENios fluorometer (excitation 485 nm, emission 510 nm).

### Cloning and mutant isolation

A stable mutation in *salA* (RL1329) was made by amplifying a 2.9 kb fragment from Rlv3841 using primers pr1310/1311 and blunt cloning into pJET1.2 blunt (Thermo Scientific), yielding pLMB607. An omega spectinomycin resistance cassette from pHP45 [27] was ligated into the EcoRI site of pLMB607 yielding pLMB608. The *salA*::ΩSp resistance cassette XhoI/Xbal from pLMB608 was cloned into the suicide vector pJQ200SK [28] forming pLMB611. This was conjugated into Rlv3841 and cells plated on AMS agar supplemented with 10% sucrose, spectinomycin, 10 mM NH_4_Cl to select for gene replacement. LMB519 was confirmed to contain the insertion by PCR mapping using primers pr1312/pOTfarForward. The double mutant (*salA*::ΩSp/*rmrA*::pK19mob) was isolated by using the general transducing phage RL38 [29] to lyse LMB519 (*salA*::ΩSp) and transduce the spectinomycin cassette into RU4314 (*rmrA*::pK19mob) as previously described [29].

Single crossovers were made independently in each gene of the *salRAB* operon (RL1328-30) of Rlv3841. Internal fragments of each gene were PCR amplified using primers RL1328_BD_F/RL1328_BD_R, RL1329_BD_F/RL1329_BD_R, and RL1330_BD_F/RL1330_BD_R, respectively. The fragments were cloned into the HindIII digested pK19 utilising BD in-fusion cloning (Clontech) to give plasmids pLMB546, pLMB492 and pLMB474 that were introduced into Rlv3841. Cells were plated on TY with neomycin (80 µg ml^−1^) to select for plasmid integration. The correct single cross-over integration in LMB455 (*salR/RL1328*::pK19mob) LMB415 (*salA/RL1329*::pK19mob) and LMB409 (*salB/RL1330*::pK19mob) were confirmed by PCR with primers RL1328_BD_map, RL1329_BD_map, RL1330_BD_map and pK19mob specific primers pK19A and B.

To construct the complementing plasmid pSal, *salRAB* was amplified using primers pr1394/pr1395 (containing KpnI and BamHI respectively on the 5′ ends) and blunt cloned into pJET1.2 blunt (Thermo Scientific). Digestion with KpnI/BamHI enabled ligation into KpnI/BamHI digested pJP2.

Transcriptional reporter plasmids pLMB557 (containing the *salR-salA* intergenic region, *salAp*) and pLMB537 (containing *salAp* as well as *salR*) were isolated by PCR amplification of fragments from Rlv3841 using primers E6_P_for/E6_P_rev and E6_RL1328_for/E6_RL1328_rev respectively. HindIII and BamHI sequences to the 5′ end of the forward and reverse primers enabled ligation of the fragments into HindIII/BamHI digested pRU1097. The correct insertions were confirmed by sequencing and conjugated into *R. leguminosarum* strains.

### Sensitivity assays

Cells were harvested from TY slopes and adjusted to an OD_600_ of 0.2. Aliquots of 100 µl were mixed into 3 ml soft TY agar (0.7%) and overlaid on TY plates. Sterile filter discs (6 mm diameter) were placed on the top agar and 5 µl of the test compound was added per disc. A two fold dilution series was used for each compound giving test concentration ranges of; Tetracycline (0.31–5 µg ml^−1^), Nalidixic acid (0.13–2 µg ml^−1^), Naringenin (0.013–5 mg ml^−1^), Geinstein (0.013–5 mg ml^−1^), Berberine (0.31–5 mg ml^−1^). Each compound at each different concentration was tested in triplicate. After 72 hours growth at 28°C the size of the zone of inhibition was measured.

### Plant growth conditions and acetylene reduction assays

Pea seeds (*Pisum sativum* cv Avola) were surface sterilised and grown in one litre pots filled with autoclaved vermiculite containing 400 ml of N-free rooting solution [24]. Plants were inoculated with 10^6^ c.f.u. and grown for 21 days in a controlled environment at 22°C under sonti-agro lights with a 16 ∶ 8 h Day ∶ Night cycle. In total there were 10 plant replicates for each strain. At harvest nodules were counted and acetylene reductions performed as previously described [30]. For each strain tested, 12 nodules were picked, surface sterilised, crushed and plated on TY medium plates. For each plate 10 individual colonies were subcultured onto Neomycin (80 µg ml^−1^) and or Spectinomycin (100 µg ml^−1^) plates to confirm the mutant genotype.

### Identification of putative multicomponent efflux pumps

Genes encoding transporter and membrane fusion proteins in Rlv3841 were identified by BLAST sequence/protein searches with the NCBI NR (http://www.ncbi.nlm.nih.gov/) and UniProtKB (http://www.uniprot.org/) databases, as well as searches with characterised systems including; *rmrAB* (MFS) from *R. etli* CFN42, *ermAB* (MFS) and *macAB* (ABC) from *Escherichia coli* K-12 MG1655, *bdeAB* (RND) from *Bradyrhizobium japonicum* USDA110 and *smeAB* (RND) of *Sinorhizobium meliloti* 1021. Motif and protein domain analysis was performed with InterPro (http://www.ebi.ac.uk/interpro/).
